# Assessment of Appetite-Regulating Hormones Provides Further Evidence of Altered Adipoinsular Axis in Early Psychosis

**DOI:** 10.3389/fpsyt.2020.00480

**Published:** 2020-05-29

**Authors:** Michał Lis, Bartłomiej Stańczykiewicz, Lilla Pawlik-Sobecka, Agnieszka Samochowiec, Artur Reginia, Błażej Misiak

**Affiliations:** ^1^Clinical Department of Internal Diseases, Endocrinology and Diabetology, The Central Clinical Hospital of the Ministry of the Interior in Warsaw, Warsaw, Poland; ^2^Department of Nervous System Diseases, Wroclaw Medical University, Wrocław, Poland; ^3^Department of Clinical Psychology, Institute of Psychology, University of Szczecin, Szczecin, Poland; ^4^Department of Psychiatry, Pomeranian Medical University, Szczecin, Poland; ^5^Department of Genetics, Wroclaw Medical University, Wrocław, Poland

**Keywords:** adipose tissue, lipid, hormone, schizophrenia, obesity

## Abstract

It has been found that antipsychotic-naïve patients with first-episode psychosis (FEP) present with impaired hormonal regulation of appetite in terms of low leptin and high insulin levels (the adipoinsular axis). These findings imply that certain intrinsic mechanisms might play a role in the development of metabolic dysregulation in early psychosis. However, clinical correlates of this phenomenon remain unknown. Moreover, these alterations have not been tested in individuals at familial high risk of psychosis (FHR-P). In this study we aimed to assess the levels of adiponectin, insulin, leptin, glucose, total cholesterol, lipoproteins and triglycerides in FEP patients, unaffected offspring of schizophrenia patients (FHR-P individuals) and healthy controls (HCs) with respect to cognitive performance and psychopathological manifestation. Participants were 35 FEP patients, 33 FHR-P individuals, and 32 HCs. Cognitive performance was assessed using the Repeatable Battery for Assessment of Neuropsychological Status (RBANS). The levels of leptin and high-density lipoproteins (HDL) were significantly lower (leptin: 10.7 ± 15.7 vs. 12.6 ± 10.1, p = 0.046, and HDL: 48.0 ± 16.9 vs. 59.8 ± 17.5 mg/dl, p = 0.007), while the levels of triglycerides and insulin were significantly higher (triglycerides: 137.4 ± 58.8 vs. 77.5 ± 33.2 mg/dl, p < 0.001, and insulin: 15.2 ± 13.1 vs. 9.6 ± 5.0 µIU/ml, p = 0.023) in FEP patients compared to HCs. These differences were significant after controlling for the effects of potential confounding factors. No significant differences in the levels of serum markers between FHR-P individuals and HCs were found. There was a significant negative correlation between the level of leptin and the RBANS language score after covarying for potential confounding factors in FEP patients (B = –0.226, p = 0.006) but not in other subgroups of participants. Our findings confirm impairment of adipoinsular axis in early psychosis. However, results of our study do not support the hypothesis that familial liability to psychosis might be associated with metabolic dysregulation. Leptin levels might be associated with cognitive deficits in FEP patients. Longitudinal studies of individuals at risk of psychosis are needed to provide insights into causal mechanisms underlying our results.

## Introduction

Cardiovascular comorbidities largely contribute to reduced life expectancy in patients with schizophrenia-spectrum disorders (1). Although environmental factors that underlie this phenomenon have been widely recognized, accumulating evidence indicates that certain intrinsic mechanisms might also be relevant. Indeed, antipsychotic-naïve or minimally-medicated patients with first-episode psychosis (FEP) present with a number of cardio-metabolic, immune and the hypothalamic-pituitary-adrenal (HPA) axis dysregulations (2). One of hypotheses beyond these observations states that psychotic disorders and cardiovascular risk factors share overlapping genetic backgrounds (3). However, the role of environmental factors (e.g., unhealthy life style or early-life stress) that act in the premorbid phase of illness cannot be excluded.

Our group performed a systematic review and meta-analysis of studies investigating the levels of appetite-regulating hormones in (FEP) patients (4). We found that patients with FEP present with increased levels of insulin and reduced levels of leptin. Subgroup analysis of antipsychotic-naïve patients confirmed these findings. No significant differences in the levels of other hormones (adiponectin, ghrelin, orexin, resistin, and visfatin) between FEP and healthy controls (HCs) were observed in this meta-analysis. Notably, studies of multiple-episode schizophrenia patients revealed increased levels of leptin and insulin (5). Although our meta-analysis did not demonstrate altered levels of adiponectin in FEP, low adiponectin levels were reported in multiple-episode schizophrenia patients (6). The authors of a recent meta-analysis of studies in this field found that low adiponectin levels in this group of patients might occur as a consequence of antipsychotic treatment (6). Notably, adiponectin is another hormone that regulates secretion of insulin. It has been found that adiponectin not only increases insulin sensitivity but it can also exert antiangiogenic, antiatherogenic and neuroprotective effects (7). Although it has been observed that adiponectin levels might be associated with cognitive performance in the general population, these observations have not been confirmed in patients with schizophrenia (8).

Leptin is a hormone released by white adipose tissue and is able to pass through the blood-brain barrier. It reduces appetite *via* interactions with receptors located in the arcuate nucleus of the hypothalamus. Moreover, leptin plays an important role in the brain development and may be responsible for learning and memory processes (9). Apart from the hypothalamus, leptin receptors are expressed by neurons of the cerebral cortex, hippocampus, basal ganglia and cerebellum (10, 11). Leptin-deficient mice not only develop extreme obesity and other components of the metabolic syndrome but also show decreased brain weight and cortical volumes (9, 12, 13). There is evidence that leptin suppresses secretion of insulin by central actions and direct effects on pancreatic cells. This peripheral regulation of insulin secretion has been named as the adipoinsular axis (14).

Insulin receptors are expressed by various cells of the central nervous system. The highest concentration of insulin receptors has been found in the olfactory bulb, cerebral cortex, hippocampus, cerebellum, and hypothalamus (15). Notably, insulin resistance has been associated with cognitive impairment in FEP patients (16). There are various mechanisms underlying the effects of central insulin signaling on cognitive performance. Indeed, central insulin plays an important role in maintaining neuronal plasticity (17). More specifically, it has been demonstrated that central insulin is involved in spatial learning and memory processes through its interactions with receptors located in the hippocampus (18). In addition, patients with type 2 diabetes are at risk of cognitive impairment across various domains, including, i.e., attention, learning, memory and executive function (19).

It should be noted that insulin resistance is not the only cardiovascular risk factor associated with cognitive impairment in schizophrenia. A recent meta-analysis revealed that a diagnosis of metabolic syndrome and its single components (hypertension, abdominal obesity, insulin resistance and dyslipidemia) are also related to cognitive deficits (20). However, this meta-analysis included only two studies that investigated the effects of dyslipidemia on cognition in schizophrenia (21, 22). These studies revealed that dyslipidemia is related to impairments of executive function, verbal memory and attention. Dyslipidemia may impact cognitive performance through various mechanisms associated with the injury of the blood-brain barrier and blood vessels as well as increased amyloid deposition (23).

Although there is evidence that subclinical indices of metabolic dysregulation, such as lipid profile disturbances, decreased leptin levels and decreased insulin sensitivity, occur in patients with FEP, it remains unknown whether these alterations are present in unaffected individuals at familial high risk of psychosis (FHR-P). Moreover, clinical correlates of metabolic dysregulation in early psychosis are yet to be established. Therefore, we aimed to compare the levels of glucose, insulin, adiponectin and leptin as well as lipid profile in FEP patients, FHR-P individuals and HCs. In addition, we investigated whether these metabolic parameters are related to psychopathological manifestation and cognitive performance.

## Material and Methods

### Participants

We enrolled 35 FEP inpatients, 33 FHR-P individuals, and 32 HCs. The group of FHR-P individuals represented unaffected offspring of patients with schizophrenia, who were diagnosed according to the ICD-10 criteria. A diagnosis of FEP was established based on the DSM-IV criteria using the Operational Criteria for Psychotic Illness (OPCRIT) checklist (24). All patients were recruited during their first inpatient treatment and they had a negative history of antipsychotic treatment before admission to the inpatient unit. The following diagnoses were established in FEP patients: schizophrenia, schizoaffective disorder, schizophreniform disorder, brief psychotic disorder, and delusional disorder. In turn, HCs were recruited through advertisements and had a negative family history of psychotic and mood disorders in first- and second-degree relatives. Participants were matched for age, sex, and parental education level (the proxy measure of socioeconomic status). The exclusion criteria were as follows: (1) comorbid neurological disorders; (2) intellectual disability; (3) physical health impairment that might affect biochemical markers measured in the study (diabetes, hypertension, coronary artery disease, autoimmune disorders, inflammatory diseases, endocrine disorders); (4) drug and/or alcohol dependence (except for nicotine) and (5) duration of antipsychotic treatment longer than 30 days. Participants were recruited in two big Polish cities (Wroclaw and Szczecin) in the time period between October, 2016 and December, 2019. The study protocol was approved by the Ethics Committee of Wroclaw Medical University (Poland) and all participants gave a written informed consent.

We used the following measures of psychopathology: the Positive and Negative Syndrome Scale (PANSS) (25), the Hamilton Depression Rating Scale (HDRS) (26), the Young Mania Rating Scale (YMRS) (27), the Global Assessment of Functioning (GAF) (28) and the Social and Occupational Assessment of Functioning (SOFAS) (28). Cognitive performance was recorded using the Repeatable Battery for Assessment of Neuropsychological Status (RBANS) (29). The RBANS includes several tests that are grouped into the following domains: immediate memory (list learning and story memory), visuospatial/constructional functions (figure copy and line orientation), language (picture naming and semantic fluency), attention (digit span and coding), and delayed memory (list recall, list recognition, story memory, and figure recall).

### Anthropometric Measures and Biochemical Parameters

All participants underwent physical examination to record the waist-to-hip ratio (WHR) and the body mass index (BMI). Blood samples were collected between 7 a.m. and 9 a.m. after overnight fasting. Subsequently, they were centrifuged to obtain serum samples. Colorimetric methods were used to determine the levels of glucose, total cholesterol, triglycerides and high-density lipoproteins (HDL) in the Konelab 60 analyzer (Argenta). The level of low-density lipoproteins (LDL) was calculated using the Friedewald formula. Electrochemiluminescence analysis was applied to measure the levels of insulin (the Cobas e411 analyzer, Roche). The levels of adiponectin and leptin were determined using the Enzyme-Linked Immunosorbent Assay (ELISA) kits.

### Statistics

Bivariate comparisons were performed using the χ^2^ test (categorical variables) and the Mann-Whitney U test or t-tests (continuous variables). Normality of data distribution was assessed using the Kolmogorov-Smirnov test. The Spearman rank correlation coefficients were used to test bivariate correlations. The analysis of covariance (ANCOVA) was performed to test for between-group differences in metabolic parameters after adjustment for age, sex, BMI, and cigarette smoking status. Similarly, linear regression analysis was performed in case of significant bivariate correlations between the measures of psychopathology or cognitive performance and metabolic parameters. Age, sex, BMI, cigarette smoking status, and chlorpromazine equivalent dosage (CPZeq) were included as covariates. Results were considered statistically significant if the p-value was less than 0.05. All analyses were conducted using the Statistical Package for Social Sciences, version 20 (SPSS Inc., Chicago, Illinois, USA).

## Results

General characteristics of the participants were shown in **Table 1**. There were no significant between-group differences in age, sex, parental education level, BMI, and WHR. As expected, cigarette smoking rates were significantly higher, while the number of education years was significantly lower, in FEP patients. Similarly, patients with FEP scored significantly lower on all domains of cognitive performance compared to HCs or FHR-P individuals (except for visuospatial/constructional abilities). In turn, FHR-P individuals had significantly lower scores of visuospatial/constructional abilities, attention and delayed memory in comparison with HCs.

**Table 1:** General characteristics of participants.

	FEP, n = 35	FHR-P, n = 33	HCs, n = 32	p (FEP vs. HCs)	p (FEP vs. FHR-P)	p (FHR-P vs. HCs)
Age, years	34.2 ± 12.5	37.3 ± 11.2	32.3 ± 8.4	0.477	0.283	0.054
Sex, M(%)	18 (51.4)	12	11	0.218	0.232	1.000
Years of education	13.7 ± 3.0	15.4 ± 3.6	15.7 ± 2.5	**0.007**	**0.044**	0.743
Paternal education, higher/other than higher (%)	7 (20.0)	3 (9.1)	7 (21.9)	1.000	0.314	0.301
Maternal education, higher/other than higher (%)	7 (20.0)	6 (18.2)	9 (28.1)	0.566	1.000	0.554
BMI, kg/m^2^	23.9 ± 4.1	24.7 ± 4.2	23.7 ± 3.1	0.876	0.400	0.281
WHR	0.9 ± 0.1	0.9 ± 0.1	0.8 ± 0.1	0.072	0.788	0.151
Cigarette smoking (%)	12 (34.3)	5 (15.2)	4 (12.5)	**0.047**	0.158	0.732
CPZeq, mg/day	347.2 ± 174.2	–	–	–	–	–
HDRS	8.8 ± 7.8	–	–	–	–	–
YMRS	2.3 ± 5.3	–	–	–	–	–
PANSS-P	15.0 ± 5.3	–	–	–	–	–
PANSS-N	19.3 ± 8.0	–	–	–	–	–
SOFAS	48.5 ± 13.1	92.4 ± 9.8	93.7 ± 6.1	**<0.001**	**<0.001**	0.519
GAF	48.0 ± 15.2	–	–	–	–	–
RBANS – immediate memory	41.7 ± 9.4	49.4 ± 6.9	49.7 ± 6.3	**<0.001**	**0.002**	0.851
RBANS – visuospatial/constructional	35.0 ± 4.5	36.1 ± 4.1	38.1 ± 2.2	**0.001**	0.291	**0.022**
RBANS – language	27.5 ± 5.8	32.1 ± 6.1	32.4 ± 6.0	**0.001**	**0.004**	0.942
RBANS – attention	50.1 ± 13.8	59.8 ± 11.7	66.4 ± 8.5	**<0.001**	**0.003**	**0.016**
RBANS – delayed memory	46.1 ± 9.4	51.1 ± 5.3	54.4 ± 4.9	**<0.001**	**0.041**	**0.009**

Significant bivariate differences (p < 0.05) were marked with bold characters.

BMI, body mass index; CPZeq, chlorpromazine equivalent dosage; FEP, first-episode psychosis; FHR-P, individuals at familial high risk of psychosis; GAF, the Global Assessment of Functioning; HCs, healthy controls; HDRS, the Hamilton Depression Rating Scale; PANSS-N, the Positive and Negative Syndrome Scale (negative symptoms subscale); PANSS-P, the Positive and Negative Syndrome Scale (positive symptoms subscale); RBANS, the Repeatable Battery for Assessment of Neuropsychological Status; SOFAS, the Social and Occupational Assessment of Functioning; WHR, waist-to-hip ratio; YMRS, the Young Mania Rating Scale.

Metabolic parameters in distinct groups of participants were presented in **Table 2**. Patients with FEP had significantly lower levels of leptin and HDL than HCs. The difference in HDL levels between FEP patients and FHR-P individuals was also significant. In turn, the levels of triglycerides and insulin were significantly higher in FEP patients compared to HCs. Moreover, patients with FEP had significantly higher levels of triglycerides than HCs. These between-group differences remained significant after covarying for age, sex, BMI, and cigarette smoking status (**Table 2**).

**Table 2:** Metabolic parameters in FEP patients, FHR-P individuals and HCs.

	FEP, n = 35	FHR-P, n = 33	HCs, n = 32	p (FEP vs. HCs)	p (FEP vs. FHR-P)	p (FHR-P vs. HCs)
Total cholesterol, mg/dl	179.6 ± 37.6	191.6 ± 39.3	183.4 ± 36.8	0.682	0.207	0.395
LDL, mg/dl	104.1 ± 35.0	112.6 ± 36.5	108.2 ± 34.8	0.633	0.335	0.627
HDL, mg/dl	48.0 ± 16.9	60.6 ± 14.1	59.8 ± 17.5	**0.007^a^**	**0.002^b^**	0.836
Triglycerides, mg/dl	137.4 ± 58.8	91.8 ± 46.3	77.5 ± 33.2	**<0.001^c^**	**0.001^d^**	0.168
Leptin, ng/ml	10.7 ± 15.7	12.6 ± 10.1	17.6 ± 19.0	**0.046^e^**	0.086	0.475
Adiponectin, µg/ml	8.2 ± 4.0	7.6 ± 5.2	7.5 ± 4.0	0.533	0.587	0.995
Glucose, mg/dl	87.5 ± 19.4	87.0 ± 17.5	85.8 ± 11.3	0.995	0.999	0.998
Insulin, µIU/ml	15.2 ± 13.1	11.2 ± 6.4	9.6 ± 5.0	**0.023^f^**	0.152	0.205

Significant bivariate differences (p < 0.05) were marked with bold characters.

^a^ANCOVA: group (FEP vs. HCs): F = 4.84, p = 0.032; BMI: F = 6.16, p = 0.016; cigarette smoking: F = 3.94, p = 0.052; age: F = 0.544, p = 0.464; sex: F = 2.71, p = 0.105.

^b^ANCOVA: group (FEP vs. FHR-P): F = 6.84, p = 0.003, BMI: F = 8.53, p = 0.005, cigarette smoking: F = 3.01, p = 0.088, age: F = 0.28, p = 0.602, sex: F = 2.32, p = 0.133.

^c^ ANCOVA: group (FEP vs. HCs): F = 25.00, p < 0.001; BMI: F = 8.92, p = 0.004; cigarette smoking: F = 0.001, p = 0.990; age: F = 0.202, p = 0.655; sex: F = 6.74, p = 0.012.

^d^ANCOVA: group (FEP vs. FHR-P): F = 15.06, p < 0.001, BMI: F = 5.56, p = 0.022, cigarette smoking: F = 0.34, p = 0.563, age: F = 0.49, p = 0.486, sex: F = 5.35, p = 0.024.

^e^ANCOVA: group (FEP vs. HCs): F = 5.04, p = 0.028; BMI: F = 13.70, p < 0.001, cigarette smoking: F = 0.559, p = 0.457; age: F < 0.001, p = 0.990; sex: F = 1.41, p = 0.239.

^f^ANCOVA: group (FEP vs. FHR-P): F = 6.45, p = 0.014; BMI: F = 1.68, p = 0.199, cigarette smoking: F = 0.197, p = 0.659; age: F = 1.05, p = 0.310, sex: F = 1.80, p = 0.185.

FEP, first-episode psychosis; FHR-P, individuals at familial high risk of psychosis; HCs, healthy controls; HDL, high-density lipoproteins; LDL, low-density lipoproteins.

There were several significant bivariate correlations between metabolic parameters, psychopathological manifestation and cognitive performance, especially in FEP patients (**Table 3**). However, only a negative correlation between leptin levels and the RBANS language score remained significant (B = −0.226, p = 0.006) in FEP patients after controlling for the effects of age (B = 0.023, p = 0.795), sex (B = 2.221, p = 0.314), BMI (B = −0.071, p = 0.817), cigarette smoking status (B = −3.771, p = 0.047) and CPZeq (B = −0.004, p = 0.358) in linear regression analysis. Other bivariate correlations between metabolic parameters, psychopathological manifestation and cognitive performance were not significant in linear regression analysis (data not shown).

**Table 3:** Correlations between metabolic parameters, psychopathological manifestation, and cognitive performance.

	TC	LDL	HDL	Triglycerides	Leptin	Adiponectin	Glucose	Insulin
**FEP:** HDRS YMRS PANSS-P PANSS-N SOFAS GAF Immediate memory Visuospatial/constructional Language Attention Delayed memory	r = 0.101 r = −0.266 r = −0.307 r = 0.047 r = 0.287 r = 0.238 r = 0.205 r = 0.111 r = −0.205 r = 0.096 r = 0.285	r = 0.213 r = −0.234 r = −0.216 r = 0.086 r = 0.181 r = 0.164 r = −0.023 r = 0.067 **r =** −**0.343^a^** r = 0.063 r = 0.139	r = −0.301 r = 0.094 r = −0.064 r = −0.179 **r = 0.340^a^** r = 0.329 **r = 0.437^a^** r = 0.148 **r = 0.440^b^** r = 0.144 r = 0.219	r = 0.391^a^ r = −0.399^a^ r = −0.175 r = 0.357^a^ r = −0.216 r = −0.246 r = −0.201 r = −0.123 **r =** −**0.484^b^** r = −0.225 r = 0.014	**r = 0.357^b^** r = −0.179 r = −0.203 r = 0.069 r = −0.125 r = −0.125 r = −0.082 r = −0.112 **r = 0.552^b^** r = 0.003 r = −0.037	r = −0.255 r = −0.037 r = 0.048 r = −0.209 r = −0.021 r = 0.004 r = 0.294 r = −0.216 r = 0.175 r = 0.038 r = 0.765	r = 0.194 r = 0.091 r = −0.061 r = −0.240 r = −0.092 r = −0.041 r = 0.243 r = −0.140 r = −0.129 r = −0.180 r = 0.003	r = 0.321 r = −0.157 r = −0.220 r = −0.112 r = −0.107 r = −0.066 r = 0.140 r = 0.007 **r =** −**0.375^a^** r = 0.005 r = 0.025
**FHR**−**P:** SOFAS Immediate memory Visuospatial/constructional Language Attention Delayed memory	r = 0.313 r = 0.224 r = 0.071 r = 0.268 r = 0.031 r = −0.189	r = 0.247 r = 0.222 r = 0.096 r = 0.274 r = 0.017 r = −0.139	r = 0.083 r = 0.225 r = 0.108 r = 0.055 r = 0.081 r = −0.043	r = 0.169 r = −0.067 r = −0.182 r = 0.154 r = −0.085 r = −0.243	r = −0.167 r = 0.267 r = −0.027 r = 0.053 r = 0.080 r = 0.141	r = 0.331 r = 0.302 r = 0.119 r = 0.087 r = 0.106 r = 0.142	r = 0.127 r = 0.029 r = −0.057 r = 0.173 r = −0.105 r = −0.270	r = −0.252 r = 0.045 r = −0.148 r = 0.091 r = 0.001 r = 0.012
**HCs:** SOFAS Immediate memory Visuospatial/constructional Language Attention Delayed memory	r = −0.112 r = −0.027 r = 0.157 r = −0.067 r = 0.245 r = −0.063	r = −0.066 r = −0.059 r = 0.189 r = −0.177 **r =** −**0.379^a^** r = −0.167	r = −0.050 r = 0.158 r = 0.055 r = 0.286 r = −0.108 r = 0.288	r = −0.014 **r =** −**0.527^b^** r = −0.115 r = −0.289 r = −0.324 r = −0.314	r = −0.021 r = 0.162 r = 0.141 r = 0.267 r = 0.316 r = 0.265	r = −0.310 **r = 0.415^a^** r = 0.061 r = 0.234 r = −0.012 r = 0.279	r = −0.008 r = −0.368 r = 0.081 r = −0.104 r = −0.040 r = −0.193	r = −0.034 r = −0.146 r = −0.012 r = −0.146 r = −0.085 r = −0.154

Significant correlations (p < 0.05) were marked with bold characters.

^a^p < 0.05, ^b^p < 0.01.

BMI, body mass index; FEP, first-episode psychosis; FHR-P, individuals at familial high risk of psychosis; GAF, the Global Assessment of Functioning; HCs, healthy controls; HDL, high-density lipoproteins; HDRS, the Hamilton Depression Rating Scale; LDL, low-density lipoproteins; PANSS-N, the Positive and Negative Syndrome Scale (negative symptoms subscale); PANSS-P, the Positive and Negative Syndrome Scale (positive symptoms subscale); SOFAS, the Social and Occupational Assessment of Functioning; TC, total cholesterol; WHR, waist-to-hip ratio; YMRS, the Young Mania Rating Scale.

## Discussion

Our results provide further evidence that impaired adipoinsular axis is an early sign of metabolic dysregulation in psychosis. These findings are consistent with results of our meta-analysis showing increased levels of insulin and decreased levels of leptin in FEP patients compared to HCs (4). Leptin is an anorexigenic hormone released by adipose tissue and suppresses the release of insulin by direct interactions with its receptors expressed by β-cells (14). Therefore, leptin deficiency can contribute to excessive release of insulin that is an adipogenic hormone. Increased storage of adipose tissue leads to overproduction of leptin and subsequent leptin resistance. Indeed, there is evidence that multiple-episode schizophrenia patients present with increased leptin levels (5). Impaired leptin signaling might also be related to the pathophysiology of psychosis. It has been found that leptin reduces dopamine neuronal firing in the mesolimbic system (30).

The observation that higher leptin levels are associated with lower RBANS scores of language performance also support the involvement of leptin in the pathophysiology of psychosis. Notably, the RBANS language score is composed of two cognitive tasks—picture naming and semantic fluency. Patients with schizophrenia show robust deficits of verbal fluency, with semantic fluency being more impaired than phonemic fluency (31). These impairments can be attributed to attenuated frontal activation (32). There is evidence that neonatal leptin deficiency reduces the frontal cortex volumes (33). Desensitization of leptin receptors in the prefronal cortex has been associated with upregulation of dopaminergic genes in this brain region (34). Another potential explanation is related to the effects of leptin on immune-inflammatory processes. Elevated levels of leptin in obesity might contribute to the release of pro-inflammatory cytokines (35). In turn, elevated levels of proinflammatory cytokines have been associated with cognitive impairment in patients with schizophrenia (36). Surprisingly, our study demonstrated a negative correlation between leptin levels and performance of the language domain. However, a cross-sectional study design does not allow to conclude regarding direction of causality. One of potential scenarios is that higher secretion of leptin is a response to neurostructural alterations of the frontal cortex and related cognitive impairment in FEP. A lack of significant correlations between the RBANS scores and leptin levels in FHR-P individuals as well as HCs further support this interpretation.

Furthermore, we demonstrated significantly lower levels of HDL as well as significantly higher levels of triglycerides in FEP patients than in HCs. These findings are also in line with those provided by recent meta-analyses of lipid profile alterations in FEP patients (37, 38). Notably, we did not confirm the hypothesis that familial liability to psychosis is related to metabolic alterations and impaired appetite regulation. However, it should be noted that our operationalization of familial liability might be insufficient to detect a significant association as we did not assess prodromal symptoms. Moreover, due to low sample size, we were not able to test our hypotheses in a subgroup of individuals meeting the criteria of at-risk mental state (genetic risk and deterioration syndrome) (39). Indeed, there is evidence that individuals at clinical high risk of psychosis show a high percentage of metabolic syndrome components prior to exposure to antipsychotic treatment (40). Moreover, it has been shown that measuring the levels of fatty acids in subjects at ultra-high risk of psychosis may improve prediction of transition to overt psychotic episode (41). Another study demonstrated that FEP patients have significantly higher levels of prolactin, fasting glucose, glycosylated hemoglobin and insulin resistance compared to individuals at clinical high risk of psychosis (42). It cannot also be excluded that results of our study simply reflect the effects of environmental factors or unhealthy lifestyle characteristics that are highly prevalent in early psychosis and include nutritional deficiencies as well as low exercise activity (43–45).

There are some important limitations of this study that need to be acknowledged. Firstly, our sample size was not large. Therefore, it cannot be excluded that our sample had insufficient power to detect the association between familial liability to psychosis and metabolic alterations. Moreover, we did not perform a detailed clinical assessment of FHR-P individuals, especially with respect to prodromal symptoms of psychosis. In light of these two limitations, we were unable to test the hypothesis whether metabolic dysregulation assessed in this study appears in individuals at clinical high risk of psychosis. Another limitation is that we cannot exclude medication effects. However, exposure to antipsychotic treatment was low in our study and linear regression analyses did not confirm a significant effect of CPZeq. It is also important to note that we did not record initial sample of individuals approached for participation and reasons for nonparticipation. Therefore, it is difficult to evaluate representativeness of our sample. Finally, a lack of longitudinal study design does not allow to establish conclusions regarding causality and temporal patterns of changes in metabolic parameters.

In summary, this study provides additional evidence of impaired adipoinsular axis, in terms of low leptin and high insulin levels, in early psychosis. Leptin levels might be related to cognitive impairment in FEP patients; however, causal mechanisms of this association need to be confirmed. Our findings provide novel insights into potential mechanisms of early metabolic disturbances and cognitive impairment in psychotic disorders. Moreover, we confirmed that FEP is associated with specific lipid profile disturbances. Longitudinal studies investigating our findings in subjects at clinical high risk of psychosis, especially in those with genetic risk and deterioration syndrome, are needed to confirm direction of causality and address limitations of our study.

## Data Availability Statement

The datasets generated for this study are available on request to the corresponding author.

## Ethics Statement

The studies involving human participants were reviewed and approved by The Ethics Committee at Wroclaw Medical University, Poland. The patients/participants provided their written informed consent to participate in this study.

## Author Contributions

ML and BM designed the study. ML, BM, BS, AS and AR were involved in recruitment of participants. LP-S measured the levels of leptin and adiponectin. BM performed data analysis. ML and BM wrote the first draft of the manuscript. All authors contributed to reviewing and editing the first draft of the manuscript.

## Funding

This study was funded from science budget resources granted for the years 2016–2019 (the Iuventus Plus grant awarded by the Ministry of Science and Higher Education, grant number: IP2015 052474).

## Conflict of Interest

The authors declare that the research was conducted in the absence of any commercial or financial relationships that could be construed as a potential conflict of interest.
